# Natural and Vaccine-Induced Acquisition of Cross-Reactive IgG-Inhibiting ICAM-1-Specific Binding of a Plasmodium falciparum PfEMP1 Subtype Associated Specifically with Cerebral Malaria

**DOI:** 10.1128/IAI.00622-17

**Published:** 2018-03-22

**Authors:** Rebecca W. Olsen, Gertrude Ecklu-Mensah, Anja Bengtsson, Michael F. Ofori, John P. A. Lusingu, Filip C. Castberg, Lars Hviid, Yvonne Adams, Anja T. R. Jensen

**Affiliations:** a Centre for Medical Parasitology at Department of Immunology and Microbiology, Faculty of Health and Medical Sciences, University of Copenhagen, Copenhagen, Denmark; b Department of Clinical Microbiology, Copenhagen University Hospital (Rigshospitalet), Copenhagen, Denmark; c Department of Infectious Diseases, Copenhagen University Hospital (Rigshospitalet), Copenhagen, Denmark; d Department of Immunology, Noguchi Memorial Institute for Medical Research, University of Ghana, Legon, Ghana; e National Institute for Medical Research, Tanga Centre, Tanga City, Tanzania; University of South Florida

**Keywords:** DBLβ cross-reactive antibodies, ICAM-1-binding motif, PfEMP1, Plasmodium falciparum, adhesion inhibition

## Abstract

Cerebral malaria (CM) is a potentially deadly outcome of Plasmodium falciparum malaria that is precipitated by sequestration of infected erythrocytes (IEs) in the brain. The adhesion of IEs to brain endothelial cells is mediated by a subtype of parasite-encoded erythrocyte membrane protein 1 (PfEMP1) that facilitates dual binding to host intercellular adhesion molecule 1 (ICAM-1) and endothelial protein receptor C (EPCR). The PfEMP1 subtype is characterized by the presence of a particular motif (DBLβ_motif) in the constituent ICAM-1-binding DBLβ domain. The rate of natural acquisition of DBLβ_motif-specific IgG antibodies and the ability to induce such antibodies by vaccination are unknown, and the aim of this study was to provide such data. We used an enzyme-linked immunosorbent assay (ELISA) to measure DBLβ-specific IgG in plasma from Ghanaian children with malaria. The ability of human immune plasma and DBLβ-specific rat antisera to inhibit the interaction between ICAM-1 and DBLβ was assessed using ELISA and *in vitro* assays of IE adhesion under flow. The acquisition of DBLβ_motif-specific IgG coincided with age-specific susceptibility to CM. Broadly cross-reactive antibodies inhibiting the interaction between ICAM-1 and DBLβ_motif domains were detectable in immune plasma and in sera of rats immunized with specific DBLβ_motif antigens. Importantly, antibodies against the DBLβ_motif inhibited ICAM-1-specific *in vitro* adhesion of erythrocytes infected by four of five P. falciparum isolates from cerebral malaria patients. We conclude that natural exposure to P. falciparum as well as immunization with specific DBLβ_motif antigens can induce cross-reactive antibodies that inhibit the interaction between ICAM-1 and a broad range of DBLβ_motif domains. These findings raise hope that a vaccine designed specifically to prevent CM is feasible.

## INTRODUCTION

Plasmodium falciparum is the major cause of the estimated 430,000 deaths due to malaria that are reported annually (1). The pathogenesis of P. falciparum is linked to sequestration of infected erythrocytes (IEs) in various tissues, which can lead to tissue-specific inflammation, circulatory obstruction, and organ dysfunction (reviewed in reference 2). IE sequestration is mediated by members of the erythrocyte membrane protein 1 (PfEMP1) family. These proteins are encoded by approximately 60 *var* genes per P. falciparum genome and are expressed on the IE surface, where they bind to a range of host receptors (reviewed in reference 3).

Despite extensive inter- and intraclonal diversity, the PfEMP1 proteins can be classified into three major groups (A, B, and C), based on *var* gene sequence and chromosomal context (4, 5). Group A is less diverse than the other groups, and expression of group A PfEMP1 proteins on the IE surface has repeatedly been linked to the development of severe malaria (6, 7). This is consistent with the restricted serological diversity of P. falciparum parasites from patients with severe malaria (8, 9). It also fits the observation that acquisition of immunity to complicated disease often precedes development of protection from uncomplicated malaria and asymptomatic parasitemia and that PfEMP1 expression is modulated by PfEMP1-specific immunity (10–12). More recently, the PfEMP1 groups have been further subdivided according to their constituent Duffy-binding-like (DBL) and cysteine-rich interdomain region (CIDR) domains, and a number of multidomain blocks, known as domain cassettes (DCs), have been identified (13–16). Three of these, DC4, DC8, and DC13, have been linked to severe malaria in children (6, 14, 17, 18). DC4 consists of three domains (DBLα_1.1/1.4_, CIDRα_1.6_, and DBLβ_3_) and defines a subfamily of group A PfEMP1 proteins that mediates binding to intercellular adhesion molecule 1 (ICAM-1) (15). IE adhesion to ICAM-1 appears associated with severe malaria, implicating DC4-specific antibodies in clinical protection, as they are acquired early in life by children living in areas where malaria is endemic and are associated with clinical protection from malaria (6, 15, 19). However, until recently, the role of IE adhesion to ICAM-1 specifically in CM has been unclear (20–24).

DC8 consists of four domains (DBLα_2_, CIDRα_1.1_, DBLβ_12_, and DBLγ_4/6_) and is found among group B/A genes, while the two-domain (DBLα_1.7_, CIDRα_1.4_) DC13 is found in some group A PfEMP1 proteins (14). Endothelial protein receptor C (EPCR) is the cognate receptor for DC8- and DC13-containing PfEMP1 proteins (25). Some studies have reported high transcript levels of *var* genes encoding EPCR-binding PfEMP1 variants in parasites from children with severe malaria, including CM, and perturbed EPCR expression in brain tissue of CM patients (26–28). While these findings point to a role for EPCR in severe malaria in general, and cerebral malaria (CM) in particular, the available evidence overall remains equivocal (29–31).

We have previously proposed that the above ambiguities may reflect that the pathogenesis of CM involves P. falciparum parasites expressing PfEMP1 capable of mediating IE adhesion to both ICAM-1 (via DBLβ) and EPCR (via CIDRα_1_) (3). A few such dual receptor-binding PfEMP1 proteins were identified shortly after, although the study did not link them to CM specifically and did not document concomitant binding to both receptors (32). However, those gaps were recently closed by our demonstration of a link between CM and group A PfEMP1 proteins capable of binding ICAM-1 and EPCR simultaneously (33). This dual receptor-binding subgroup of PfEMP1 proteins is characterized by an EPCR-binding CIDRα_1_ domain followed immediately by a DBLβ domain featuring a specific ICAM-1-binding motif (DBLβ_motif domain) (33).

The rate of natural acquisition of IgG against the DBLβ_motif associated specifically with CM and the ability to induce such antibodies by vaccination are both unknown. The current study was therefore designed to investigate whether cross-reactive IgG antibodies specific for DBLβ_motif domains and/or their ICAM-1-binding motif are acquired following natural exposure to P. falciparum parasites and whether ICAM-1 adhesion inhibitory antibodies can be induced by immunization with specific DBLβ_motif proteins and with peptides representing the ICAM-1-binding motif therein. Confirmation of these hypotheses would support the feasibility of developing a vaccine designed specifically to prevent CM.

## RESULTS

### Delayed acquisition of IgG to ICAM-1-binding group A-type DBLβ domains.

Group A PfEMP1 proteins that contain a DBLβ domain with the motif I(V/L)X_3_N(E)GG(P/A)XYX_27_GPPX_3_H (DBLβ_motif domains) (where X represents an unknown amino acid) can bind to the host endothelial receptor ICAM-1 and always feature a neighboring CIDRα_1_ domain that enables concomitant binding to another endothelial receptor, EPCR (33). Expression of these dual receptor-binding PfEMP1 proteins is associated with CM, which is a major cause of mortality and severe morbidity among African children. The age at which most CM cases occurs varies with transmission intensity but generally occurs later than the peak prevalence of parasitemia and malaria-related severe anemia. DBLβ domains present in group A PfEMP1 proteins, but without the above motif (DBLβ_nonmotif domains), do not bind ICAM-1 and are less conserved in the C terminus (33).

**FIG 1:**
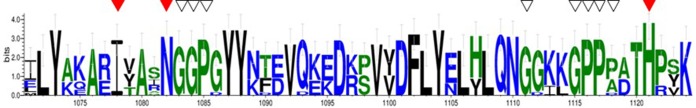
Sequence logo showing the ICAM-1-binding motif (as defined in reference 33) of DBLβ domains 1 to 15 used in the present study (Table 3). Residues that are critical for the direct interaction with ICAM-1 (red triangles) or for the architecture of the ICAM-1 binding (white triangles) are indicated. The group A PfEMP1 ICAM-1-binding motif was identified by Lennartz et al. (33).

**FIG 2:**
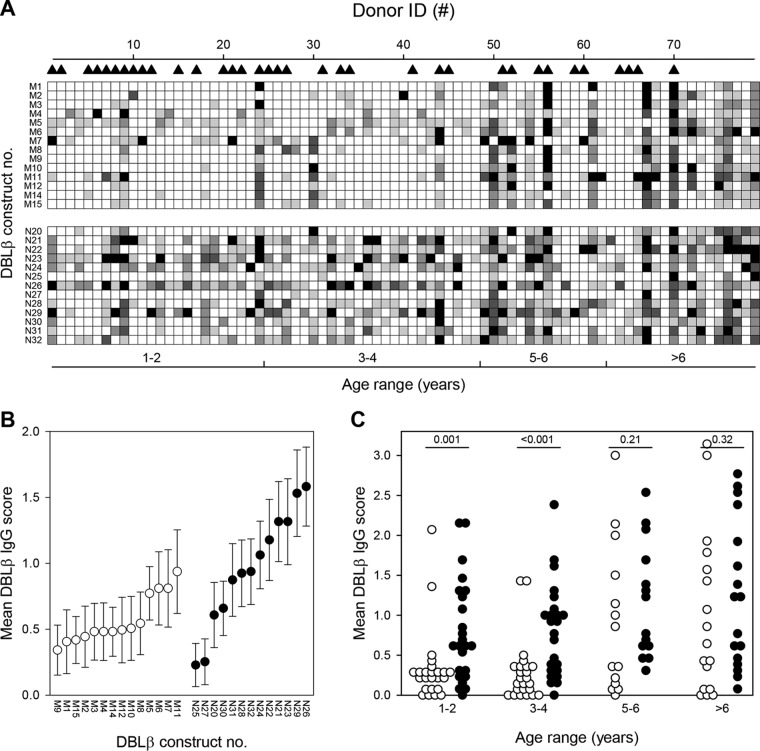
Plasma levels of IgG with specificity for P. falciparum DBLβ domains. Samples were obtained from 79 Ghanaian children with either severe (▲) or nonsevere P. falciparum malaria. (A) Levels (ELISA units [EU]) in plasma from individual children (columns) of IgG antibodies specific for individual group A DBLβ domains (rows) containing (DBLβ_motif; M1 to M12, M14, and M15) or not containing (DBLβ_nonmotif; N20 to N32; lower half) the ICAM-1-binding motif identified by Lennartz et al. (33). Shading indicates IgG level score: black, score of 4 (>100 EU); dark gray, score of 3 (76 to 100 EU); gray, score of 2 (51 to 75 EU); light gray, score of 1 (26 to 50 EU); white, score of 0 (0 to 25 EU). The DBLβ domain numbers correspond to the numbers in Table 3. Danish controls (*n* = 25) did not react with any of the domains (data not shown). (B) The means of IgG level scores (defined as described for panel A) of individual DBLβ domains that contain (motif; ○) or do not contain (nonmotif; ●) the ICAM-1-binding motif. Error bars indicate 95% confidence intervals. DBLβ domain numbering is as described for panel A. (C) The means of IgG level scores (defined as described for panel A) of individual children for IgG specific for DBLβ_motif (○) and DBLβ_nonmotif (●) domains. The statistical significance (Mann-Whitney rank sum test) of pairwise comparisons is shown along the top of the panel.

We first used ELISA to measure the levels of IgG with specificity for 14 recombinant DBLβ_motif domains (M1 to M12, M14, and M15) (Fig. 1) and 13 nonmotif DBLβ domains (N20 to N32) in plasma from 79 Ghanaian children with different clinical presentations of P. falciparum malaria (Table 1). The antibody reactivity to all these group A PfEMP1 proteins differed substantially among the children (Fig. 2A) and also among the different DBLβ domains (Fig. 2B). Overall, the plasma levels of IgG increased with age (*P_r_* < 0.001, where *r* is Pearson's product moment correlation), with levels of IgG specific for DBLβ_motif proteins being generally lower than those of DBLβ_nonmotif-specific IgG (*P_T_* < 0.001, where *T* is the Kruskal-Wallis one-way analysis of variance on ranks). However, the latter difference was due mainly to low IgG recognition of the DBLβ_motif among the younger age groups (≤4 years of age) (Fig. 2C). Thus, the levels of IgG specific for the DBLβ_motif were significantly lower than the DBLβ_nonmotif-specific IgG levels among children aged 1 to 2 years and 3 to 4 years (*P_T_* ≤ 0.001), but not in the two older age classes considered (5 to 6 years and >6 years; *P_T_* ≥ 0.21).

**TABLE 1:** Clinical characteristics of Ghanaian study participants contributing plasma

Characteristic	Value^*a*^ for participants with:							
	Severe malaria (*n* = 35)				Uncomplicated malaria (*n* = 44)			
Age group (range of yrs)	1–2 (*n* = 16)	3–4 (*n* = 9)	5–6 (*n* = 6)	>6 (*n* = 4)	1–2 (*n* = 8)	3–4 (*n* = 15)	5–6 (*n* = 8)	>6 (*n* = 13)
Age (yrs)	2.2 (1.9; 2.7)	3.4 (3.2; 4.3)	5.5 (5.3; 6.6)	7.8 (7.5; 8.2)	2.5 (1.8; 2.7)	3.9 (3.4; 4.4)	5.9 (5.3; 6.9)	9.2 (8.1; 10.8)
Blantyre coma score	5.0 (5.0; 5.0)	5.0 (4.3; 5.0)	5.0 (4.3; 5.0)	4.0 (3.0; 5.0)	5.0 (5.0; 5.0)	5.0 (5.0; 5.0)	5.0 (5.0; 5.0)	5.0 (5.0; 5.0)
Hemoglobin (g/dl)	9.6 (5.6; 10.8)	6.3 (3.1; 9.6)	10.3 (8.8; 10.9)	10.8 (7.0; 12.0)	9.6 (8.9; 10.3)	9.6 (6.9; 10.8)	9.5 (8,4; 11.9)	11.4 (10.6; 11.7)
Parasites/μl (×1,000)	41.5 (14.3; 171.0)	147.3 (4.3; 210.8)	123.2 (2.9; 204.9)	114.5 (56.3; 152.6)	8.3 (0.4; 26.0)	18.0 (6.3; 62.0)	19.7 (3.2; 71.2)	14.7 (1.6; 109.9)

a Values are medians (25th percentile; 75th percentile). None of the participants donating plasma was diagnosed with cerebral malaria.

The overall plasma levels of IgG specific for DBLβ_motif domains as well as DBLβ_nonmotif domains were similar in patients with severe and uncomplicated malaria (*P_U_* = 0.4 in both cases, where *U* is the Mann-Whitney test for intergroup differences), while levels of DBLβ_nonmotif domain-specific IgG were lower in the children with severe malaria than in the patients with uncomplicated disease (scores, 7 and 12.5; *P_U_* = 0.02, respectively).

Taken together, these results indicate that DBLβ_motif and DBLβ_nonmotif proteins are similarly immunogenic but that DBLβ_motif-specific IgG is acquired later in life than DBLβ_nonmotif-specific IgG.

### Immunization with DBLβ_motif antigens induces cross-reactive, neutralizing IgG.

**FIG 3:**
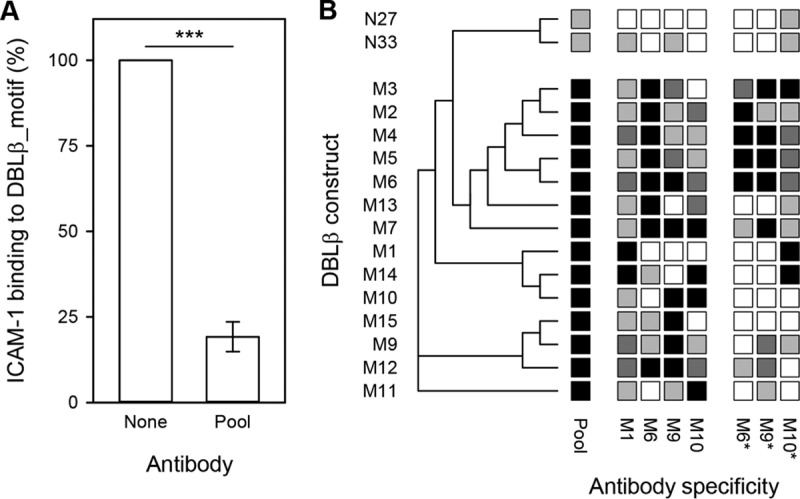
Ability of DBLβ_motif-specific IgG to inhibit binding of ICAM-1 to DBLβ domains. (A) Inhibition of ICAM-1 binding by pooled immune plasma from 10 of the study children (pool). ***, *P_F_* < 0.001. (B) Rat antisera raised against DBLβ_motif antigens tested against recombinant proteins containing (M1 to M7 and M9 to M15) or not containing (N27 and N33) the DBLβ_motif. Shading indicates the degree of inhibition: black, >75%; dark gray, 50 to 75%; gray, 20 to 50%; white, <20%. The DBLβ domain numbers and antiserum specificities correspond to the data in Table 3. Data using a pool of rat antisera (M1, M6, M9, M10) are also shown (pool). Antisera marked with asterisks were affinity purified on a peptide (M6pep) representing the binding motif in PFD1235w_DBLβ_D4 prior to assaying. Three independent experiments were done (three technical replicates/assay). A sequence-distance tree illustrating the relatedness of the different domains is shown along the left edge of the panel.

Plasma from clinically immune individuals living in areas of stable and intense transmission of P. falciparum parasites can inhibit the interaction between ICAM-1 and a range of DBLβ_motif domains (15, 33). In this study, a pool of plasma from 10 of the children (selected for reactivity with DBLβ_motif domains [Fig. 2A and Table 1] and plasma availability) inhibited ICAM-1 binding to DBLβ_motif protein M9 (Fig. 3A). These data suggest the presence of neutralizing IgG antibodies capable of recognizing multiple DBLβ_motif-containing PfEMP1 variants (cross-reactive IgG). If such antibodies could be induced by vaccination, it would increase the feasibility of developing a broadly protective PfEMP1-based vaccine against cerebral malaria. However, our data might also reflect the presence of many different variant-specific IgG specificities, where each antibody specificity is capable of inhibiting the binding of ICAM-1 to only a particular DBLβ_motif domain variant (a broad repertoire of IgG with narrow specificity). It is inherently difficult to distinguish between these two alternatives in naturally acquired immunity. Nevertheless, truly cross-reactive PfEMP1-specific human antibodies have previously been demonstrated in a study employing naturally acquired monoclonal IgG specific for the VAR2CSA-type PfEMP1 involved in the pathogenesis of placental malaria (34).

To investigate the relative importance of the above nonexclusive alternatives, to further assess the functional significance of DBLβ_motif in acquired immunity, and to make a first step toward PfEMP1-based vaccination specifically against cerebral malaria, we immunized four rats with DBLβ_motif proteins M1, M6, M9, and M10, respectively (see Table 3; see also Fig. S1 in the supplemental material). We used an enzyme-linked immunosorbent assay (ELISA) to test the ability of the antisera to inhibit the binding of ICAM-1 to 14 DBLβ_motif proteins (M1 to M7 and M9 to M15) and two ICAM-1-binding DBLβ_nonmotif proteins (N27 and N33). Each of the four DBLβ_motif-specific antisera inhibited binding of ICAM-1 to most of the DBLβ_motif proteins by more than 50% but had little effect on ICAM-1-binding to DBLβ_nonmotif domains (Fig. 3B). When pooled, the DBLβ_motif-specific antisera strongly inhibited (>75%) binding of ICAM-1 to all DBLβ_motif domains, with much less effect (<50%) on ICAM-1-binding to the DBLβ_nonmotif domains (Fig. 3B). We next affinity purified IgG from three of the rat antisera, using M6pep, to evaluate the involvement of IgG directly targeting the ICAM-1-binding region in the above-described inhibition. The purified M6pep-specific IgG generally inhibited ICAM-1 binding to the same degree as the antisera (Fig. 3B), with strong correlation between the antiserum and motif-specific IgG data for M6 (Spearman's rank order correlation [*r_s_*] = 0.78; *P* < 0.001).

Overall, these data indicate that immunization with single DBLβ_motif antigens can induce cross-reactive IgG that inhibits binding of ICAM-1 to the homologous, as well as a broad range of heterologous, DBLβ_motif domains.

### DBLβ_motif-specific IgG is broadly inhibitory of IE adhesion to ICAM-1 under physiologic flow.

**FIG 4:**
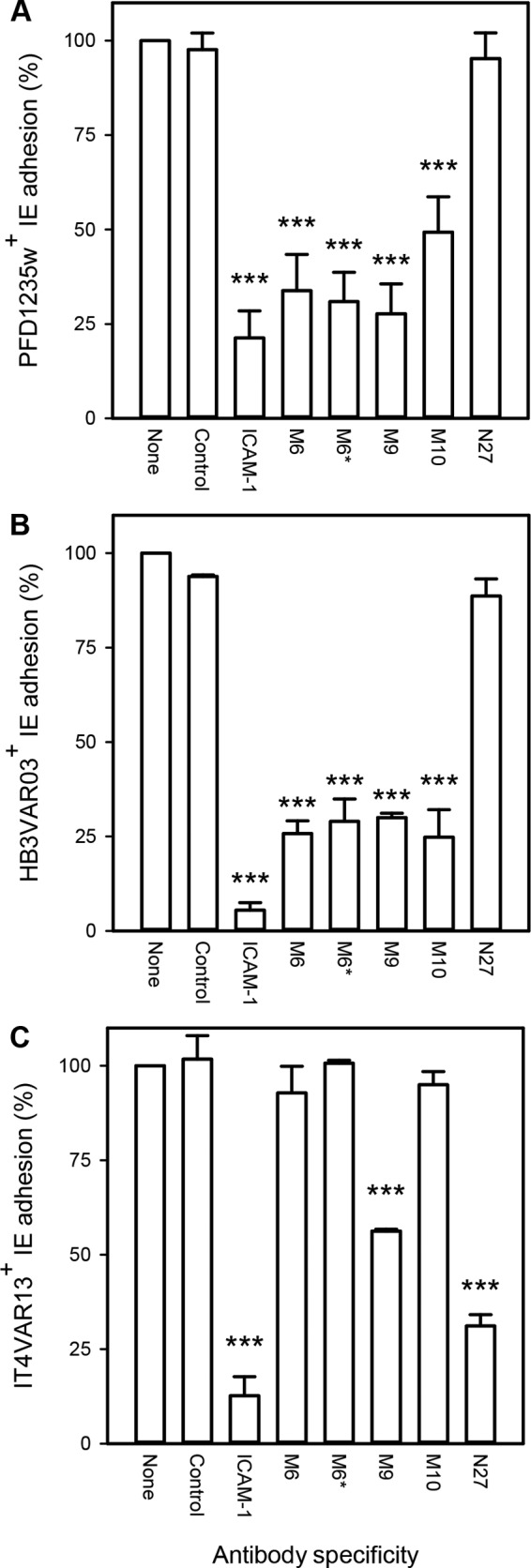
DBLβ-specific antibody-mediated inhibition of adhesion of IEs to ICAM-1 under physiologic shear stress, relative to that of control without antibody (none). (A) IEs expressing PFD1235w; (B) IEs expressing HB3VAR03; (C) IEs expressing IT4VAR13. The specificities of the DBLβ antibodies correspond to the data in Table 3. The antiserum marked with an asterisk was affinity purified on a peptide (M6pep) representing the binding motif in PFD1235w DBLβ_D4 prior to assaying. An ICAM-1-specific neutralizing antibody (ICAM-1) and an irrelevant rat anti-IgG (Sigma-Aldrich) were included as positive and negative controls, respectively. Fewer than 0.25 IEs/mm^2^ bound to uncoated channels. Means (bars) and standard deviations (error bars) of the results of at least three independent experiments in triplicate are shown. Statistically significant reductions relative to adhesion in the absence of antibody (−) are indicated above the bars (**, *P_F_* < 0.01; ***, *P_F_* < 0.001). See Table S1 in the supplemental material for raw data.

The ability of neutralizing IgG to interfere with receptor-specific IE sequestration *in vivo* likely depends on the characteristics of the involved PfEMP1 proteins *per se*, on their expression on the IE surface, and on the shear forces at the anatomical location of the interaction of IEs with host endothelium. We have previously shown that naturally acquired IgG can inhibit ICAM-1-specific adhesion of erythrocytes infected by P. falciparum expressing DBLβ_motif-containing PfEMP1 (33). To test if such antibodies could also be elicited by immunization with recombinant PfEMP1 proteins containing the DBLβ_motif, we tested the ability of DBLβ_motif-specific IgG to inhibit the adhesion of IEs to ICAM-1 in an *in vitro* assay simulating physiologic flow conditions (35). M6-specific IgG significantly inhibited ICAM-1-specific adhesion of IEs expressing the homologous PfEMP1 (PFD1235w; *P_F_* < 0.001, where *F* is one-way analysis of variance) (Fig. 4A) or a heterologous PfEMP1 (HB3VAR03; *P_F_* < 0.001) (Fig. 4B). This was also the case for IgG specific for M9 and M10 (see Table 3) and IgG purified on the ICAM-1-binding motif in M6 (M6pep; *P_F_* < 0.001). The M9-specific antibodies also affected ICAM-1-specific adhesion of IEs expressing IT4VAR13, a group B PfEMP1 protein that binds ICAM-1 but does not contain the DBLβ_motif (see reference 33) (Fig. 4C). Conversely, an antiserum to the ICAM-1-binding domain in IT4VAR13 (N27) inhibited ICAM-1-specific adhesion of the homologous IEs (Fig. 4C) but had no effect on ICAM-1-specific adhesion of IEs expressing the DBLβ_motif-containing PfEMP1 protein PFD1235w (Fig. 4A) or HB3VAR03 (Fig. 4B).

We conclude from these experiments that immunization with DBLβ_motif antigens induces cross-reactive IgG antibodies that inhibit ICAM-1-specific adhesion of IEs that express a variety of PfEMP1 proteins containing DBLβ_motif domains. The inhibition of binding of native PfEMP1 protein to ICAM-1 under conditions of flow thus mirrors that observed with recombinant proteins in ELISA.

### Immunization with peptides representing the ICAM-1-binding region induces antibodies broadly inhibiting the binding of recombinant and native DBLβ_motif domains to ICAM-1.

**FIG 5:**
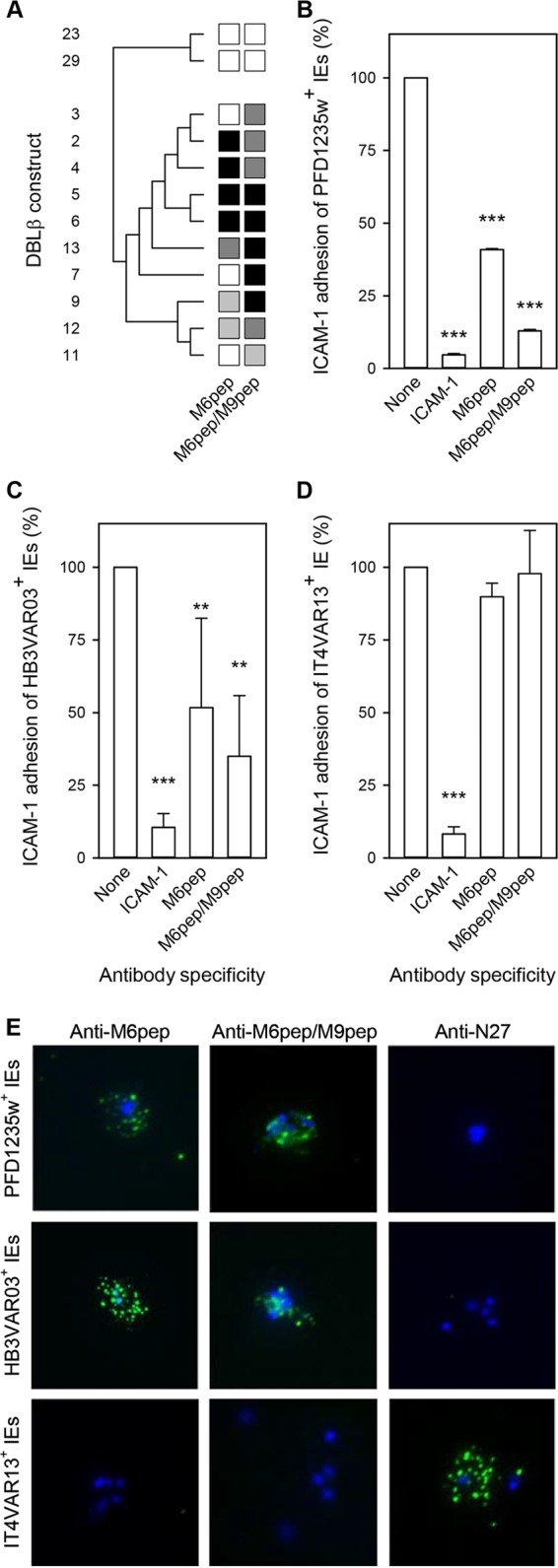
Ability of rat antisera to the ICAM-1-binding motif in DBLβ_motif domains to inhibit binding of recombinant DBLβ domains to ICAM-1. (A) Antisera from rats immunized with M6pep or with both M6pep and M9pep tested against recombinant DBLβ_motif domains (M2 to M7, M9, and M11 to M13) and DBLβ_nonmotif domains (N27 and N33). Shading, DBLβ domain numbers, and antiserum specificities are as described in the legend to Fig. 3B. (B to D) Inhibition by the same antisera of ICAM-1-specific adhesion of PFD1235w-positive IEs (B), HB3VAR03-positive IEs (C), and IT4VAR13-positive IEs (D) under physiologic shear stress. The statistical significance of the reductions is indicated as described in the legend to Fig. 4. Three independent experiments were done (with three technical replicates in each). Fewer than 0.25 IEs/mm^2^ bound to uncoated channels were observed. (E) Immunofluorescence of representative IEs with surface expression of PFD1235w (top row), HB3VAR03 (center row), and IT4VAR13 (bottom row) and labeled by sera from rats immunized with M6pep only (left column) or with both M6pep and M9pep (center column), or by a rat antiserum to N27 (right column). See Table S2 in the supplemental material for raw data.

The results above suggested that IgG targeting the ICAM-1-binding region in DBLβ_motif domains is of particular importance for inhibiting the binding of group A dual receptor-binding PfEMP1 to ICAM-1. This interpretation is further supported by our recent data showing that DBLβ_motif-purified antibodies from naturally infected humans and experimentally vaccinated animals inhibit ICAM-1-specific adhesion of IEs expressing the DBLβ_motif-containing PfEMP1 protein PFD1235w (33). To assess directly if inhibitory and cross-reactive antibodies could be elicited by peptide immunization, we immunized rats with peptides representing the ICAM-1-binding region in DBLβ_motif domains (M6pep and M9pep) and tested their ability to inhibit binding of ICAM-1 to DBLβ_motif domains. Antisera from rats immunized with M6pep only, or with M6pep and M9pep, were broadly inhibitory of the binding of ICAM-1 to 10 DBLβ_motif domains (M2 to M7, M9, and M11 to M13). The peptide antisera did not affect binding to two ICAM-1-binding DBLβ_nonmotif domains (N27 and N33) (Fig. 5A). Experiments assessing the ability of the antisera to inhibit the adhesion of IEs to ICAM-1 under flow corroborated these findings. Thus, both of the above-mentioned antisera (M6pep and M6pep/M9pep) significantly inhibited the adhesion of IEs expressing the DBLβ_motif-containing PfEMP1 proteins PFD1235w (Fig. 5B) (expressing M6 native protein) and HB3VAR03 (Fig. 5C) (expressing M8 native protein) but had no effect on IEs expressing IT4VAR13 (expressing N27 native protein), which does not contain a DBLβ_motif domain (Fig. 5D). Furthermore, the single- and dual-peptide antisera yielded immunofluorescence patterns typical of IgG reacting with IE surface-expressed PfEMP1 when tested against IEs expressing either HB3VAR03 or PFD1235w but did not label IEs expressing IT4VAR13 (Fig. 5E). In contrast, the IEs expressing IT4VAR13 were labeled by an IT4VAR13-specific antiserum but not by the single- and dual-peptide antisera (Fig. 5E).

**FIG 6:**
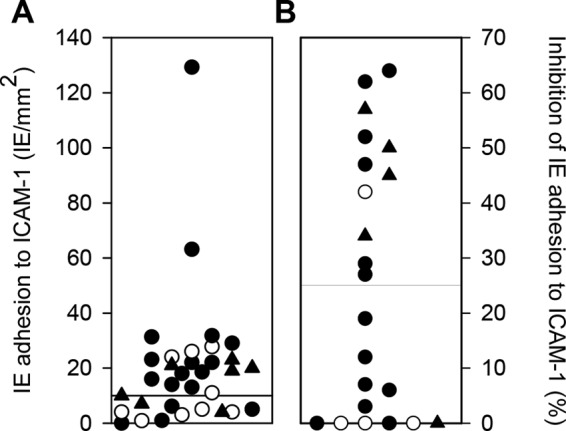
ICAM-1-specific adhesion of erythrocytes infected by patient P. falciparum isolates and inhibition of ICAM-1-adhering IEs by M6pep/M9pep-specific antibody. (A) Adhesion of 33 patient isolates to ICAM-1 under physiologic flow; (B) antibody-mediated inhibition (>25%) of ICAM-1-specific IE adhesion among the 22 patient isolates adhering (≥10 adherent IEs/mm^2^) to ICAM-1 under physiologic flow. The isolates were tested in one experiment with five technical replicates. Fewer than 0.25 IEs/mm^2^ bound to uncoated channels. Isolates from patients with uncomplicated malaria (○), cerebral malaria (▲), and noncerebral severe disease (●) are indicated in both panels.

Finally, we assessed the ability of erythrocytes infected by 33 primary P. falciparum isolates (Table 2) from Ghana (*n* = 14) and Tanzania (*n* = 19) to adhere to ICAM-1 under flow. We also tested the ability of pooled rat antiserum to M6pep and M9pep to inhibit the adhesion of ICAM-1-adhering isolates. Twenty-two of the isolates (3 from children with uncomplicated malaria, 14 from patients with severe malaria, and 5 from children with cerebral malaria) showed adhesion of IEs to ICAM-1 (Fig. 6A). The adhesion of 11 of these isolates (1 from a child with uncomplicated malaria, 6 from children with severe malaria, and 4 from children with cerebral malaria) was inhibited (>25%) by the anti-peptide serum pool (Fig. 6B).

**TABLE 2:** Clinical characteristics of Ghanaian and Tanzanian study participants contributing P. falciparum parasite isolates

Characteristic	Value^*a*^ for participants with:							
	Severe malaria (*n* = 24)				Uncomplicated malaria (*n* = 9)			
Age group (range of yrs)	<1 (*n* = 3)	1–2 (*n* = 11)	3–4 (*n* = 7)	≥5 (*n* = 3)	<1 (*n* = 1)	1–2 (*n* = 2)	3–4 (*n* = 4)	≥5 (*n* = 2)
Age (yrs)	0.9 (0.89; 0.95)	2.0 (1.67; 2.53)	4.01 (3.47; 4.78)	7.4 (5.56; 7.68)	0.36	2.50, 2.76	3.6 (3.07; 4.58)	6.4, 11.3
Blantyre coma score	1.0 (0.0; 5.0)	5.0 (2.0; 5.0)	5.0 (2.0; 5.0)	3.0 (2.0; 3.0)	5.0	5.0, 5.0	5.0 (5.0; 5.0)	5.0, 5.0
Hemoglobin (g/dl)	7.8 (4.2; 8.2)	4.7 (4.4; 6.1)	8.6 (3.8; 10.8)	10.5 (6.1; 11.8)	11.8	12.0, 12.3	8.6 (6.9; 10.2)	12.5, 11.7
Parasites/μl (×1,000)	64.8 (4.0; 165.4)	74.8 (33.2; 194.5)	182.9 (42.2; 456.2)	63.3 (49.2; 77.5)	91.0	77.5, 70.5	58.9 (18.7; 133.4)	87.4, 13.7

a Values are medians (25th percentile; 75th percentile), except for the columns with data from two patients, which show the actual values. The severe malaria group includes patients with cerebral malaria (*n* = 7), severe malarial anemia (*n* = 11), and hyperparasitemia, multiple convulsions, and/or respiratory distress (*n* = 9).

We conclude that immunization with linear peptides that represent only the ICAM-1-binding region of specific DBLβ_motif domains can induce cross-reactive antibodies that are capable of inhibiting the binding of ICAM-1 to a range of recombinant and native, IE-expressed DBLβ_motif domains. Importantly, the motif antibody inhibits the binding of four of five P. falciparum isolates from cerebral malaria patients.

## DISCUSSION

Cerebral malaria (CM) is one of the most severe complications of P. falciparum malaria and a leading cause of mortality (reviewed in reference 36). PfEMP1-mediated adhesion of IEs to the endothelial receptors ICAM-1 and EPCR have both repeatedly been implicated in the pathogenesis of severe malaria (15, 25, 33). However, a specific and direct link to the development of CM has been missing until recently, when we identified a sequence motif in PfEMP1 proteins associated specifically with the development of CM (33). This ICAM-1-binding motif (DBLβ_motif) (Fig. 1) is found in some group A PfEMP1 proteins (15), immediately downstream of an EPCR-binding CIDRα domain (25). In the present study, we set out to study the acquisition of DBLβ_motif-specific IgG following natural exposure and whether DBLβ_motif-specific antibodies induced by vaccination are cross-reactive and inhibit adhesion to ICAM-1.

In areas with stable transmission of these parasites, substantial protective immunity to malaria is acquired during childhood, first to severe complications and later to clinical disease. As a consequence, adults are largely protected from malaria in such areas, although sterile immunity is rarely, if ever, achieved. This sequence appears to be the consequence of an ordered acquisition of antibodies to a relatively conserved set of PfEMP1 proteins associated with severe disease, followed by antibodies to a large and diverse set of PfEMP1 proteins associated with uncomplicated malaria and asymptomatic parasitemia. Where transmission is very intense, serious and fatal malaria episodes are markedly concentrated during the first few years of life, mainly as severe malarial anemia. CM, in contrast, is rare (37). Where endemicity is lower, CM tends to be seen more often but mainly among children some years older than those that succumb to severe malarial anemia. Together these findings suggest that discrete PfEMP1 subsets are involved in severe malaria with and without cerebral involvement and perhaps even that toddlers are relatively resistant to CM for nonimmunologic reasons. This fits our demonstration here that although DBLβ domains are generally immunogenic following natural exposure, acquisition of DBLβ_motif-specific IgG occurs later than that of DBLβ_nonmotif-specific IgG (Fig. 2) and coincides with the age bracket in which the incidence of CM peaks under transmission intensities comparable to those in our study area (38, 39).

The clinical significance of acquisition of PfEMP1-specific antibodies is thought to involve their ability to interfere with sequestration of IEs in various tissues (15, 40). A particularly thoroughly investigated example is the role of anti-adhesion antibodies in the acquisition of protective immunity to placental P. falciparum malaria, caused by accumulation of IEs in the intervillous spaces (41, 42). Placental IE sequestration is mediated by a particular group of PfEMP1 (VAR2CSA) binding to oncofetal chondroitin sulfate A (43, 44), and clinical trials of vaccines based on the VAR2CSA adhesive epitope and aimed to protect against this important cause of prenatal and infant morbidity and mortality are under way. It appears that DBLβ_motif-specific IgG can inhibit IE adhesion to ICAM-1 in a similar way (Fig. 3A) (15, 33). Here, we demonstrate that this inhibition can be mediated by genuinely cross-reactive antibodies, as opposed to a broad repertoire of IgG species, each with narrow specificity for a single or very few DBLβ_motif sequences (Fig. 3). This finding is of significance, since naturally acquired protection from malaria is generally believed to be the consequence of the accumulation of a broad repertoire of a large number of antibody specificities (9, 12, 45). Such broadly reactive IgG can inhibit the adhesion of erythrocytes infected with parasites isolated from patients with severe malaria (6 of 13 isolates) and cerebral malaria (4 of 5 isolates) under physiologic flow conditions (Fig. 6). Furthermore, such IgG can be induced by peptides (M6pep and M9pep) representing just the core element of the DBLβ_motif that mediates the binding to ICAM-1 (Fig. 5 and 6).

Approximately half the children with acute P. falciparum malaria in our study had severe disease, according to the WHO criteria (46), but we did not observe any significant differences in plasma levels of DBLβ_motif-specific IgG in children with or without severe disease. This may be related to the fact that due to the low prevalence in the area none of the study children had CM (Table 1). It is doubtful that a relationship between DBLβ_motif-specific IgG and clinical presentation of acutely ill malaria patients would be apparent, even if we had been able to include plasma samples from CM patients, due to the unavoidable variation in time between infection and presentation to hospital. It is plausible that only very large, and preferably longitudinal, studies would have the power required to document such relationships in semi-immune, naturally infected individuals.

In conclusion, our study demonstrates that CM-related DBLβ domains are immunogenic following natural exposure and that acquisition of DBLβ_domain-specific IgG coincides with the age where CM has its peak prevalence in areas of moderate but stable P. falciparum transmission. Furthermore, we show that immunization with such domains in addition to peptides representing the minimal ICAM-1-binding region can induce IgG that can inhibit PfEMP1 binding to ICAM-1 and neutralize IE adhesion under physiologic flow. Importantly, these antibodies broadly neutralized the adhesion of erythrocytes infected by parasites isolated from four of five children with cerebral malaria. Together, these findings raise hopes that development of a vaccine specifically against CM may be possible, despite the notorious polymorphism and intraclonal diversity of the PfEMP1 family (recently reviewed in references 3 and 47).

## MATERIALS AND METHODS

### Plasma and parasite samples.

Plasma samples (*n* = 79) for the present study were collected in 2014 at Hohoe Municipality Hospital in the Volta Region of Ghana from children with acute malaria (Table 1) (33, 48).

P. falciparum parasites were collected at this hospital (*n* = 14) and at the Korogwe District Hospital (*n* = 19) in Korogwe District in northeastern Tanzania (14). Clinical manifestations of malaria were classified according to the definitions and associated criteria of the World Health Organization. Patients were categorized as having cerebral malaria (CM; *n* = 7) if they had a positive blood smear of the asexual form of P. falciparum and unrousable coma (Blantyre coma score [BCS] ≤ 2) with exclusion of other causes of coma and severe illness. Patients were categorized as having severe malarial anemia (SA; *n* = 12) if the hemoglobin level was <5 g/dl and the BCS was >2. Patients were classified as having severe malaria other than SA and CM if they presented with hyperparasitemia (>250,000 parasites/μl), multiple convulsions (>2 episodes in 24 h), respiratory distress (i.e., rapid, deep, and labored breathing), or combinations of these symptoms. Patients with uncomplicated malaria (UM; *n* = 48) had fewer than 250,000 parasites/μl.

The study was approved by the Ethical Review Committee of the Ghana Health Services (file GHS-ERC 08/05/14) and by the National Ethical Review Committee of the National Institute for Medical Research, Tanzania (NIMR/HQ/R.8a/Vol.IX/559). A pool of plasma from P. falciparum-exposed Tanzanian individuals (49) and 25 nonexposed Danish individuals were used as positive and negative controls, respectively. Long-term *in vitro* culture-adapted and fully sequenced parasite clones 3D7, HB3, and IT4 were also studied.

### Recombinant proteins.

The genes encoding the DBLβ domains used were amplified from genomic DNA or produced as synthetic genes (http://eurofins.dk) (Table 3). Amplicons were subcloned into a modified pET15b vector and expressed as His-tagged proteins in Escherichia coli Shuffle C3030 cells (New England BioLabs) as described previously (15). All the proteins were purified (see Fig. S1 in the supplemental material) by immobilized metal ion affinity chromatography using HisTrap HP 1-ml columns (GE Healthcare) and are referred to by the designations listed in Table 3.

**TABLE 3:** Recombinant proteins used in the study

Domain ID	Genome	PfEMP1	Domain subtype^*a*^	Binds ICAM-1^*b*^	Group^*c*^	Gene source^*i*^
M1	3D7	PF11_0521^*d*^	DBLβ3_D4	Yes^*j*^	A	
M2	BM048	JF712902	DBLβ3_D4	Yes^*j*^	A	
M3	BM066	JF712903	DBLβ3_D4	Yes^*j*^	A	
M4	BM021	JF712900	DBLβ3_D4	Yes^*j*^	A	
M5	BM057	JN037695	DBLβ3_D4	Yes^*j*^	A	
M6	3D7	PFD1235w^*e*^	DBLβ3_D4	Yes^*j*^	A	
M7	MN35	KJ866957	DBLβ3_D4	Yes^*j*^	A	
M8	HB3	VAR03	DBLβ3_D4	Yes^*j*^	A	
M9	Dd2	VAR32^*f*^	DBLβ1_D4	Yes^*j*^	A	
M10	MN56	KM364031	DBLβ1_D4	Yes^*j*^	A	
M11	A4395	KJ866958	DBLβ3	Yes^*j*^	A	
M12	1914	AFJ66668	DBLβ1_D4	Yes^*j*^	A	
M13	BM028	JF712901	DBLβ3_D4	Yes^*j*^	A	
M14		KM364033	DBLβ3	Yes^*j*^	A	
M15	MN062	KF984156	DBLβ1_D4	Yes^*j*^	A	
N21		CDO61797		No^*k*^	A	Synthetic gene (Eurofins Genomics)
N22		CDO63496		No^*k*^	A	Synthetic gene (Eurofins Genomics)
N23	Dd2	VAR25	DBLβ11_D4	No^*j*^	A	
N24	HB3	VAR1CSA	DBLβ11_D4	No^*j*^	A	
N25	3D7	PF13_0003	DBLβ9_D8	No^*j*^	A	
N26	A4393	KJ866959	DBLβ3	No^*j*^	A	
N27	IT4	IT4VAR13^*g*^	DBLβ3_D4	Yes^*j*^,^*l*^	B	
N28	1983	JQ691647	DBLβ3_D4	No^*j*^	A	
N29	MN35	KM364034	DBLβ6	No^*j*^	A	
N30	Dd2	VAR52	DBLβ7_D4	No^*j*^	A	
N31	HB3	VAR01	DBLβ7_D4	No^*j*^	A	
N32	1983	JQ691649	DBLβ6_D4	No^*j*^	A	
N33	IT4	IT4VAR16^*h*^	DBLβ5_D4	Yes^*j*^,^*l*^	B	Genomic DNA using forward and reverse primers: 5′-ATCCCGGGTGTGCTGAACCTAATGGTAG-3′ and 5′-ATGCGGCCGCTACAAGCACACGCATCATC-3′

a Nomenclature as described in reference 15.

b “Yes” indicates DBLβ_motif domains that bind ICAM-1 (M1 to M15, N27, and N33); “no” indicates domains that do not bind ICAM-1 (the remainder).

c All domains were group A except for two group B DBLβ domains as indicated (N27 and N33).

d Also known as PF3D7_1150400.

e Also known as PF3D7_0425800.

f Also known as KOB85388.

g Also known as ABM88750.

h Also known as AAS89259.

i From genomic DNA, using previously described primers (15, 33), except where indicated.

j Data from reference 33.

k Unpublished data.

l Data from reference 61.

Recombinant Fc-tagged ICAM-1 was expressed in HEK293 cells and purified on a HiTrap Protein G HP column (http://www3.gehealthcare.dk/) as described previously (50).

### DBLβ-specific antisera.

We generated rat antisera to recombinant proteins M1, M6, M9, M10, N27, and N33 (Table 3) and to two synthetic peptides (Schafer-N) that corresponded to the ICAM-1-binding motifs in M6 (M6pep, LYAKARIVASNGGPGYYNTEVQKKDRSVYDFLYELHLQNGGKKGPPPATHPYKSVNTRDKRDATDDTTP) and M9 (M9pep, LYKEAEIYARNGGPGYYNTEVQKEDKPVVDFLYELHLQNGGKKGPPAATHPSKSVTTRVKRDTTVDTPS). M1, M6, M9, and M10 were selected from different branches of the previously published phylogenetic tree of DBLβ domains, to represent dual ICAM-1- and EPCR-binding group A PfEMP1 proteins (33). In a similar way, N27 and N33 were chosen as random examples of ICAM-1-binding group B PfEMP1 proteins.

In each case, Wistar rats were immunized with the antigen (25 μg) in Freund's incomplete adjuvant, followed by two booster vaccinations 2 weeks apart (15 μg/boost). Blood was collected 2 weeks after the last immunization. All animal procedures were approved by the Danish Animal Procedures Committee (“Dyreforsøgstilsynet”) as described in permit no. 2013-15-2934-00920, and all experiments were done according to the guidelines described in Danish act LBK 1306 (23 November 2007) and BEK 1273 (12 December 2005).

### IgG purification.

IgG antibodies specific for M6 and M6pep were affinity purified from rat antisera as described previously (51). In brief, M6 and M6pep (1 mg/ml) were dialyzed overnight against coupling buffer and coupled to HiTrap normal human serum (NHS)-activated HP columns, as described by the manufacturer (GE Healthcare). Antisera were diluted 1:1 in phosphate-buffered saline (PBS) and affinity purified on the columns, followed by elution of bound IgG in low-pH buffer (glycine-HCl, pH 2.75) and pH adjustment by Tris-HCl (1 M, pH 9.0).

### Measurements of DBLβ-specific IgG levels.

MaxiSorp microtiter plates (Sigma-Aldrich) were coated with recombinant DBLβ domains (50 μl; 5 μg/ml) as described previously (15). Plasma samples (diluted 1:100 in blocking buffer) were incubated (50 μl/well, 1 h, room temperature) in duplicate wells. The plates were washed (PBS plus 1% Triton X-100), and bound antibody was detected with horseradish peroxidase (HRP)-conjugated anti-human IgG (1:3,000 in blocking buffer) (Agilent). After incubation (1 h) and washing as described above, bound detection antibody was detected using *o*-phenylenediamine dihydrochloride (OPD) tablets, according to the manufacturer's instructions (Agilent). The optical density (OD) values were read at 490 nm using a VERSAmax microplate reader (Molecular Devices). Antibody reactivity was expressed in arbitrary ELISA units (EU) calculated by the equation (OD_sample_ − OD_background_)/(OD_positive control_ − OD_background_) × 100 (52).

### Measurements of antibody-mediated inhibition of DBLβ binding to ICAM-1.

Inhibition of recombinant DBLβ domain binding to ICAM-1 by human immune plasma and rat antisera was measured by ELISA. In brief, wells of MaxiSorp plates were coated with recombinant ICAM-1 (50) (50 μl/well, 2 or 4 μg/ml, 0.1 M glycine-HCl buffer, pH 2.75) by incubation overnight (4°C) and blocked with blocking buffer (1 h, room temperature). His-tagged DBLβ proteins (0.5 to 16 μg/ml final concentration) were mixed with immune plasma or antisera (1:5 final concentration) or purified IgG (10 μg/ml final concentration) and added to duplicate wells (1 h, room temperature). The plates were washed, and binding was detected using HRP-conjugated anti-penta-His antibody (Qiagen) as described above. All antisera were prescreened by ELISA to verify the absence of His tag-reactive antibodies.

### *In vitro* culture and antibody selection of P. falciparum parasites.

The P. falciparum clones 3D7, HB3, and IT4 were maintained in long-term *in vitro* cultures and antibody selected for IE surface expression of specific PfEMP1 proteins as described previously (15, 51). In brief, we used the human monoclonal IgG antibody AB01 to select 3D7 IEs for the expression of PFD1235w (53). HB3 IEs were similarly selected for surface expression of VAR03 using a rat antiserum against M8, and IT4 IEs were selected for expression of VAR13 by a rat antiserum against N27 (33). In all cases, expression of the required PfEMP1 on the surface of mature IEs purified on a VarioMACS separator was monitored by flow cytometry using PfEMP1-specific antisera, essentially as described previously (51, 54). Only cultures with >60% antibody-labeled IEs were used.

In addition, primary isolates of P. falciparum parasites from 33 of the above-mentioned malaria patients were cultured *in vitro* for up to 28 days (median and 25th and 75th percentile, 8, 2.5, and 13 days, respectively) in Albumax (10%) (ThermoFisher Scientific), supplemented with NHS (2%), essentially as described previously (55). The genotypic identity of the isolates was routinely verified by genotyping as described previously (56), and Mycoplasma infection was regularly excluded using the MycoAlert Mycoplasma detection kit (Lonza) according to the manufacturer's instructions.

### Adhesion of IEs to ICAM-1 under physiological flow *in vitro*.

Microslides (VI^0.1^) (Ibidi) were coated with recombinant Fc-tagged ICAM-1 protein (50) (50 μg/ml, 4°C, overnight) and blocked using PBS plus 2% bovine serum albumin (BSA). Parasite suspensions, adjusted to 3% parasitemia and 1% hematocrit in RPMI 1640 supplemented with 2% NHS (pH 7.2), were flowed over the coated slides (5 min) at a shear stress of 1 dyn/cm^2^ as described previously (35). The numbers of bound IE/mm^2^ in five separate fields were counted, using a Leica inverted phase-contrast microscope (×20 magnification). To assess the capacity of affinity-purified DBLβ-specific IgG to inhibit adhesion, IEs selected for expression of particular PfEMP1 variants were preincubated with the purified IgG (15 min, room temperature). The receptor specificity of the IE adhesion observed was verified by preincubating the ICAM-1-coated flow channels with an ICAM-1-specific antibody (40 μg/ml, clone 15.2; AbD Serotec).

The inhibition of ICAM-1-adhering erythrocytes (≥10 adherent IEs/mm^2^) infected with primary P. falciparum field isolates was tested using pooled rat antisera (1:100 dilution) to two peptides representing the ICAM-1-binding motifs in M6 (M6pep) and M9 (M9pep). A minimum of three independent experiments were completed for each of the tested laboratory clones (3D7, HB3, IT4), whereas each of the field isolates was tested in one experiment with five technical replicates. All assays were deidentified for the operator.

### Immunofluorescence microscopy of IEs labeled with PfEMP1-specific antibodies.

Immunofluorescence microscopy was done essentially as described previously (57). Briefly, aliquots (50 μl) of erythrocytes infected by parasites expressing PFD1235w, HB3VAR03, or IT4VAR13 were adjusted to 5% parasitemia and resuspended in PBS containing 1% Ig-free BSA (Sigma-Aldrich). Antisera were added (1:50 dilution), and the suspensions were incubated on ice (1 h). Following three washes, cells were resuspended and labeled with anti-rat–fluorescein isothiocyanate (FITC) secondary antibody (1:500) and incubated on ice for 1 h. Cells were washed three times, and thin smears were made. Nuclei were visualized by adding 5 μl ProLong Gold antifade mountant (ThermoFisher Scientific) prior to the addition of coverslips. Immunofluorescence was visualized with a Nikon Eclipse TE2000 microscope equipped with a ×63 objective.

### Bioinformatics.

Multiple alignments of DBLβ domains known to bind ICAM-1 were made using MUSCLE v.3.7 software (58), and sequence distance trees were made with MEGA software (59). A WebLogo 3 sequence logo (60) of the ICAM-1-binding motif was generated based on alignment of the included DBLβ_motif domains (Table 3) with the consensus motif I(V/L)X_3_N(E)GG(P/A)XYX_27_GPPX_3_H (15, 33).

### Statistics.

We used Pearson's product moment correlation (*r*) or Spearman's rank-order correlation (*r_s_*) to evaluate parameter association and one-way analysis of variance (*F*), the Kruskal-Wallis one-way analysis of variance on ranks (*T*), and the Mann-Whitney test (*U*) to test for intergroup differences.

## Supplementary Material

Supplemental material
