# Supplementary material for: Natural and Vaccine-Induced Acquisition of Cross-Reactive IgG-Inhibiting ICAM-1-Specific Binding of a Plasmodium falciparum PfEMP1 Subtype Associated Specifically with Cerebral Malaria
Source: Infect Immun. 2018 Mar 22;86(4):e00622-17. doi: 10.1128/IAI.00622-17 (PMC5865037; doi:10.1128/IAI.00622-17)
Supplement: Supplemental material [file supp_86_4_e00622-17__index.html]

Natural and Vaccine-Induced Acquisition of Cross-Reactive IgG-Inhibiting ICAM-1-Specific Binding of a Plasmodium falciparum PfEMP1 Subtype Associated Specifically with Cerebral Malaria — Supplemental material 

# Natural and Vaccine-Induced Acquisition of Cross-Reactive IgG-Inhibiting ICAM-1-Specific Binding of a Plasmodium falciparum PfEMP1 Subtype Associated Specifically with Cerebral Malaria

## Supplemental material

- Supplemental file 1 -

  Fig. S1. DBLβ protein domains expressed and purified from *E. coli*. Table S1. *P. falciparum* IE (PFD1235w, HB3VAR03, IT4VAR13) flow-based ICAM-1 adhesion. Table S2. *P. falciparum* IE (PFD1235w, HB3VAR03, IT4VAR13) flow-based ICAM-1 adhesion.

  PDF, 540K
