# Supplementary material for: Natural and Vaccine-Induced Acquisition of Cross-Reactive IgG-Inhibiting ICAM-1-Specific Binding of a Plasmodium falciparum PfEMP1 Subtype Associated Specifically with Cerebral Malaria
Source: Infect Immun. 2018 Mar 22;86(4):e00622-17. doi: 10.1128/IAI.00622-17 (PMC5865037; doi:10.1128/IAI.00622-17)
Supplement: Supplemental material [file IAI.00622-17_zii999092369s1.pdf]

Supplemental Material

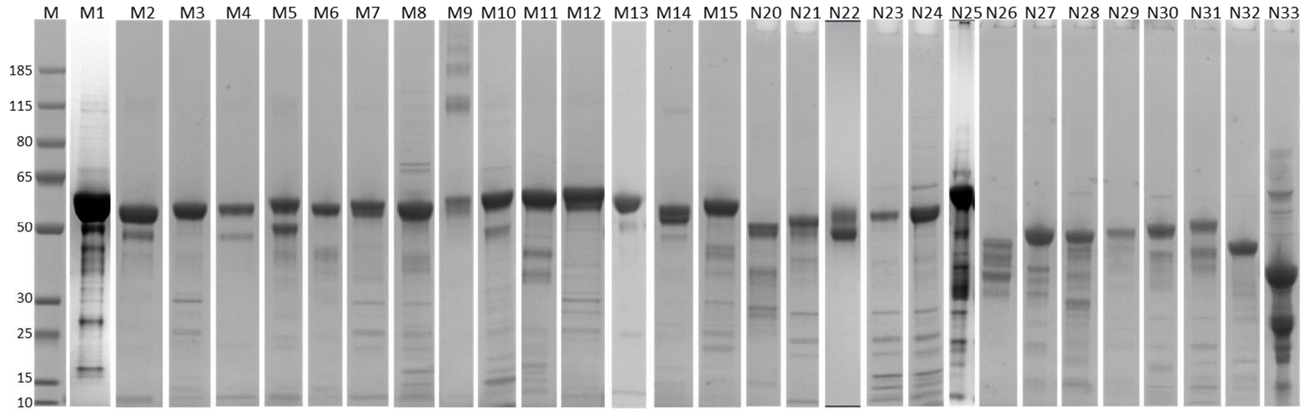

FIG S1 DBLβ protein domains expressed and purified from *E. coli*. Sodium dodecyl sulfate polyacrylamide gel electrophoresis (SDS-PAGE) gel electrophoresis of protein marker (lane M) and DBLβ protein domains (M1-M12, N20-N33. For identity, refer to Table 3). Samples were reduced using DTT.

TABLE S1A *P. falciparum* IE (PFD1235w, HB3VAR03, IT4VAR13) flow based ICAM-1 adhesion

PFD1235w^{+} infected erythrocytes

	(number bound per mm^2)**								percentage (%) binding							
Ab	None	Ctrl	ICAM1	M6	M6*	M9	M10	N27	None	Ctrl	ICAM1	M6	M6*	M9	M10	N27
Exp1	26.5	26.0	8.5	4.3	2.4	11.5	17.5	24.0	100	98.1	32.1	9.1	16.0	43.4	66.0	90.6
Exp2	10.8	11.3	2.6	2.9		2.0	5.2	11.7	100	104.9	24.1		26.5	18.5	48.2	108.6
Exp3	14.5	13.0	1.13	3.2		3.1	4.9	12.5	100	89.9	7.8		22.1	21.2	33.6	86.6
Exp4	22.8		4.8		3.0				100		21.1	13.2				
Exp5	16.0	9.5	4.2		4.6				100	59.4	26.3	28.8				
avg	18.1	15.0	4.2	3.4	3.3	5.5	9.2	16.1	100	88.1	22.3	17.0	21.6	27.7	49.3	95.3
SD	6.4	7.5	2.8	0.7	1.1	5.2	7.2	6.9	0	20.1	9.0	10.4	5.3	13.7	16.2	11.7
SEM	2.9	3.8	1.2	0.4	0.7	3.0	4.2	4.0	0	10.1	4.0	6.0	3.0	7.9	9.4	6.8

**Raw data for flow experiments in Fig. 4. *affinity purified antibody

TABLE S1B

HB3VAR03^{+} infected erythrocytes

	(number bound per mm^2)**								percentage (%) binding							
Ab	None	Ctrl	ICAM1	M6	M6*	M9	M10	N27	None	Ctrl	ICAM1	M6	M6*	M9	M10	N27
Exp1	14.3	13.3	4.3	4.1	4.3	4.3	5.5	11.6	100	93.2	2.8	30.3	30.3	30.3	38.2	81.1
Exp2	21.1	19.9	5.9	4.	3.8	3.7	2.8	18.6	100	94.0	4.1	17.7	18.0	27.8	13.3	88.0
Exp3	27.5	25.9	8.7	8.1	10.6	7.6	6.3	26.6	100	94.40	9.5	27.7	38.6	31.8	22.8	96.8
avg	21.0	1.3	6.3	5.4	6.2	5.2	4.8	18.9	100	93.9	5.5	25.2	29.0	30.0	24.8	88.7
SD	6.6	1.1	2.2	2.3	3.8	2.1	1.8	7.5	0.0	0.5	3.2	6.0	9.3	1.8	11.3	7.0
SEM	3.8	0.7	1.3	1.4	2.2	1.2	1.0	4.3	0	0.3	2.0	3.9	6.0	1.2	7.3	4.6

**Raw data for flow experiments in Fig. 4. *affinity purified antibody

TABLE S1C

	IT4VAR13$^+$ infected erythrocytes															
	(number bound per mm^2)**								percentage (%) binding							
Ab	None	Ctrl	ICAM1	M6	M6*	M9	M10	N27	None	Ctrl	ICAM1	M6	M6*	M9	M10	N27
Exp1	91.5	83.8	15.3	72.3	91.0	50.7	86.2	29.7	100	91.6	16.8	94.2	99.5	55.4	88.0	32.4
Exp2	84.8	85.5	15.8	82.0	86.7	48.5	87.5	30.2	100	100.8	18.7	103.1	102.2	57.2	91.4	35.6
Exp3	42.7	48.3	1.1	43.9	42.9	24.0	37.9	10.9	100	112.9	2.5	88.6	100.3	56.2	45.9	25.4
avg	73.0	72.5	10.7	66.1	73.5	41.1	70.5	23.6	100	101.8	12.6	95.3	100.6	56.2	75.1	31.1
SD	26.5	21.0	8.4	19.8	26.6	14.8	28.3	11.0	0	10.7	8.8	12.3	1.4	0.9	6.1	5.2
SEM	15.3	12.1	4.8	11.4	15.4	8.6	16.3	6.4	0	6.2	5.1	7.1	0.8	0.5	3.5	3.0

**Raw data for flow experiments in Fig. 4. *affinity purified antibody

TABLE S2A *P. falciparum* IE (PFD1235w, HB3VAR03, IT4VAR13) flow based ICAM-1 adhesion

	PFD1235w+ infected erythrocytes							
	(number bound per mm^2)*				percentage (%) binding			
Ab	None	ICAM1	M6	M6/M9	None	ICAM1	M6	M6/M9
Exp1	19.3	0.8	7.8	2.4	100	4.2	34.9	12.5
Exp2	16.2	0.8	6.7	2.2	100	5.2	50.4	13.4
Exp3	17.7	1.1	7.3	2.5	100	6.1	41.4	14.1
avg	17.7	0.9	7.3	2.4	100	5.1	42.2	13.3
SD	1.6	0.9	0.6	0.2	0	5.1	7.8	4.0
SEM	0.9	0.1	0.3	0.1	0	0.6	7.8	2.2

*Raw data for flow experiments in Fig. 5.

TABLE S2B

	HB3VAR03+ infected erythrocytes							
	(number bound per mm^2)*				percentage (%) binding			
Ab	None	ICAM-1	M6	M6/M9	None	ICAM-1	M6	M6/M9
Exp1	13.5	0.9	11.5	8.0	100	6.5	85.0	59.0
Exp2	21.9	3.4	10.0	5.4	100	15.7	45.7	24.8
Exp3	25.2	2.3	6.2	5.3	100	9.3	24.5	21.2
avg	20.2	2.2	9.2	6.2	100	10.5	51.7	35.0
SD	6.0	1.3	2.7	1.5	0	4.7	30.7	20.9
SEM	3.5	0.7	1.6	0.9	0	2.7	17.7	12.1

*Raw data for flow experiments in Fig. 5.

TABLE S2C

	IT4VAR13+ infected erythrocytes							
	(number bound per mm²)*				percentage (%) binding			
Ab	None	ICAM-1	M6	M6/M9	None	ICAM-1	M6	M6/M9
Exp1	40.5	4.2	46.4	41.8	100	10.3	114.4	103.1
Exp2	41.3	3.7	35.4	44.8	100	8.8	85.9	108.6
Exp3	44.1	2.4	41.0	40.3	100	5.4	93.2	91.5
avg	41.9	3.4	40.9	42.3	100	8.2	97.8	101.1
SD	1.9	0.9	5.5	2.3	0	2.5	14.8	8.7
SEM	1.1	0.5	3.2	1.3	0	1.4	8.6	5.0

*Raw data for flow experiments in Fig. 5.
